# Metabolomics profile in diabetes and blood glucose regulation: a systematic review

**DOI:** 10.3389/fnut.2026.1929989

**Published:** 2026-09-11

**Authors:** Aishat O. Adejoh, Queen S. Abbah, John O. Onuh

**Affiliations:** Department of Food and Nutritional Sciences, College of Agriculture, Environment and Nutrition Science, Tuskegee University, Tuskegee, AL, United States

**Keywords:** biomarkers, diabetes mellitus, glucose metabolism, lipid metabolism, metabolomics

## Abstract

**Introduction:**

Diabetes mellitus is a chronic, non-communicable metabolic disorder of global public health concern. Many studies utilize metabolomics to profile metabolites in individuals with diabetes. However, these studies have shown inconsistencies in identifying metabolite signatures associated with the disease without clearly defining how it influences glucose homeostasis. Discrepancies in analytical platforms and biomarker validation limit clinical translation of metabolomic biomarkers. This review aims to integrate existing evidence on metabolomic profiles in diabetes, and address challenges related to biomarker validation and method standardization.

**Methods:**

The review was conducted in adherence with PRISMA 2020 guidelines, from PubMed and Google Scholar databases. Peer-reviewed English-language studies published between 2010 and 2026 were retrieved using predefined and related keywords. Following thorough screening, 188 studies were included. The review synthesizes diverse metabolomics evidence into a unified model, establishing a link between metabolic disruptions and regulation of blood glucose.

**Results:**

Across the studies, a consistent metabolic pattern was identified, characterized by alterations in amino acid metabolism notably branched chain and aromatic amino acids, lipid metabolism including ceramides, sphingolipids, fatty acids, and central energy pathways like the TCA cycle.

**Discussion:**

Rather than being present as isolated biomarkers, they form interconnected networks that collectively influence insulin sensitivity, β-cell function, and hepatic glucose. Also, while advancement of LC-MS, GC-MS, and NMR-based metabolomics led to expansion in the identification of biomarkers, variability in experimental design and inadequate method standardization persist as constraint in reproducibility. Metabolomics offers strong framework to understand diabetes and improve early diagnosis, risk prediction, and precision glucose management, but requires better standardization.

## 1 Introduction

### 1.1 Definition of diabetes mellitus and types

Diabetes mellitus (DM) is a group of metabolic, non-communicable disorders characterized by high glucose levels in the blood. The high level of glucose results from insulin resistance, increased glucagon secretion, or β-cell dysfunction which leads to insufficient insulin production (1). The β-cells are located within a group of cells known as the islets of Langerhans and is produced in the pancreas (2). DM is regarded as a lifestyle, metabolic/endocrine disease that is characterized by diverse and in many cases, severe complications, which leaves a huge cost burden for treatment and management (3, 4). These complications include hypertension, heart attack, stroke, retinopathy, nephropathy, neuropathy, amputations, and death in extreme cases (3). For these reasons, timely detection and effective management and treatment are of the utmost significance. According to Reilly et al. (5), diet and other factors, such as genetics, are crucial in the development of the disease. Alam et al. (6) highlighted that there is a stronger tendency of developing the disease for people with hyperglycemia in their genetic predisposition.

The condition is classified into three major distinct categories. These includes Type-1 diabetes (T1D), Type-2 diabetes (T2D), and gestational diabetes (GD) (7). Each category is marked by its own unique pathophysiological mechanisms and risk factors. Hypertension, aging, obesity, and genetic predisposition are among the key risk factors. In T1D, the body’s immune system attacks and destroys the beta cells in the pancreas and over time this could lead to inadequate insulin production in the body (2). It is the most common type of diabetes affecting children and adolescents (8). Certain environmental factors, such as diet, infection, and family history, are considered risk factors in the occurrence of T1D. T2D is much more common and primarily involves progressively impaired glucose regulation due to impaired pancreatic beta cell function and insulin resistance (2). It is marked by elevated blood glucose levels, resulting from insulin resistance or impaired insulin secretion. The prevalence of the condition is higher among individuals who consume excessive amounts of alcohol and maintain diets that are deficient in nutrients and high in calories from refined sugars and grains. It is also higher among individuals who are obese or have a genetic predisposition which leads to the development of its complications, such as renal and cardiovascular diseases (CVD), retinopathy, and in extreme cases, death. GD usually occurs in pregnant women and is marked by the onset of hyperglycemia in pregnant women who were not previously diagnosed with diabetes. Most times, the condition typically resolves postpartum (9). The underlying cause of gestational GD is mostly attributed to the inability of the maternal pancreas to adequately keep up with the elevated insulin demand which plays a key role in glucose metabolism and regulation during pregnancy (9, 10).

According to recent reports, the prevalence of DM is estimated to be affecting approximately 463 million people worldwide (9.3%) as of 2019. This figure is expected to rise to 10.3 and 10.9% by 2030 and 2045, respectively (11). Alam et al. (6) reported an estimated rise of up to 552 million people by 2030 in the absence of better control and cure by the international diabetes federation. Diabetes has been identified as one of the top 10 causes of death globally (12). At present, India, China, and the USA account for the highest number of patients with this disease. A recent analysis projected a global cost for the treatment and management of diabetes and its complications to 966 billion USD in 2021, with a prediction of 1,054 billion USD rise by 2045.

Due to a rise in both cost and economic impact of the disease, there is a significant interest in ways to prevent it, especially for T2D (13). Individuals with prediabetes have a higher chance of advancing to T2D, which is usually characterized with multiple etiological factors that are associated with metabolic syndrome (MetS) (14). It is marked by dysfunction in glucose metabolism, organ inflammation linked to hyperglycemia, an increase in free fatty acids, triglycerides, and methylglyoxal, as well as a deficiency of endogenous antioxidants such as superoxide dismutase, catalase, ceruloplasmin, and antioxidant nutrients (14). Free radical stress and inflammation are recognized as the primary risk factors for T2D and its complications (15). According to Ferrannini et al. (16), α-hydroxybutyrate (α-HB), an organic acid that mediates amino acid catabolism and glutathione synthesis in the tricarboxylic acid (TCA) cycle, and linoleoyl-glycerophosphocholine (L-GPC) are biomarkers of T2D. They have direct association with glucose intolerance as well as insulin sensitivity and signaling in the body.

The human body is a complex system that is comprised of multiple interconnected components and systems which work together to ensure optimal physiological functions (17). These systems and their pathways are intricately linked to form a delicate balance within the body. The fundamental principle underlying these processes is the ability of these organs to maintain a constant and stable internal environment (18). A disruption in this homeostasis can result in the onset of an injury or a pathological state in multiple organs (19). The identification of metabolic pathways and biomarkers that are indicative of early changes is not only conducive to a comprehensive understanding of the etiology of T2D. It also has the potential to establish a theoretical foundation for the development of early diagnostic methods, risk prediction models, and prevention strategies for the disease (15). Lately, the rapid progress in metabolomics has opened fresh insights into the pathogenesis of diabetes in the human body. This modern technique analyzes many small molecules or metabolites in blood, cells, or tissues and helps researchers uncover important markers associated with the disease (20). Despite the considerable progress made in metabolomics research, existing findings remain varied with respect to the integration of metabolomic alterations with the physiological mechanisms regulating blood glucose homeostasis. Many studies have identified metabolite signatures associated with insulin resistance, β-cell dysfunction, and diabetes progression without clearly synchronizing the mechanism by which these metabolic disruptions affects glucose homeostasis. In addition, discrepancies in analytical platforms, and biomarker validation have impeded the clinical translation of metabolomic biomarkers for early diagnosis and personalized treatment due to challenges in biomarker validation and standardization. The objective of this review is to integrate existing evidence on metabolomic profiles in diabetes and blood glucose regulation, reveal key metabolic pathways linking metabolite alterations to glycemic imbalance. The study also hopes to assess the potential for early diagnosis and personalized diabetes management, while addressing challenges related to biomarker validation, method standardization, and clinical translation into practice.

## 2 Methodology and data collection

### 2.1 Literature search

Articles considered for publication in this review were carefully sorted out from peer-reviewed research publications. They were limited to articles written in English-written that were published between 2010 and 2026 in line with the PRISMA 2020 systematic review guidelines. Two databases (Google Scholar and PubMed) were utilized for the search, with search criteria modified based on review objective suitability. The databases were last searched on the 3rd of July 2026 for this review.

### 2.2 Search criteria and search strings

Articles relevant to this work were assessed by inputting keywords, phrases, or both into the search space of Google Scholar and PubMed databases and setting the advanced search criteria for the articles. Some of the search words or phrases utilized for the advanced search include but not limited to “Diabetes metabolomics profile,” “metabolites,” “insulin resistance,” “glycemic control,” “diabetes mellitus,” “glucose regulation,” “metabolomics biomarkers,” “early detection of diabetes,” “analytical tools for diabetes metabolites,” and “metabolomics and diabetes.” The “AND” and “OR” Boolean operators were used to combine the words or phrases in the search filter as well. Other search criteria utilized for the study are open-access full-text articles, books, and systematic peer-reviewed articles. However, publication names, article types, or author names were not included as filters, but the cited works were vetted to ascertain their level of significance to the study objectives.

### 2.3 Search process

After selecting articles using specific search phrases, an assessment of abstracts, titles, and keywords were used to help eliminate recurrent and irrelevant works. Articles that met the selection criteria were kept, following a thorough assessment of how relevant they are to the study, by reviewing their full texts. The present article includes all studies that met the eligibility criteria.

### 2.4 Inclusion and exclusion criteria

The inclusion criteria for the study included original research articles that investigated the application of metabolomics approaches in diabetes. Studies that involved animal or human participants diagnosed with diabetes, or classified as prediabetic or at risk, those that used a metabolomics approach, both targeted and untargeted on any established analytical platform (NMR spectroscopy, GC-MS, LC-MS, CE-MS, or similar) to identify, characterize, or validate metabolic biomarkers were included. Included in the study also were original, peer-reviewed research articles that were published in English between January 2010 and July 2026. Those that provided sufficient information on study design, sample type, analytical methods, and metabolomics data analysis were also included.

The exclusion criteria involved studies that were review articles, conference abstracts, editorials, or case reports. Also, studies were excluded if they did not include metabolomics analysis. Those not written in English, duplicates, those that exclusively focused on other omics approaches as well as those whose full text were not available were also excluded.

### 2.5 Classification of the relevant publications

The information gathered from the cited articles was categorized into headings, subheadings, pictorial representations, and tables. This review article is categorized into the following subheadings: definition and overview of metabolomics; analytical platforms used in metabolomics; targeted and untargeted metabolomics; sample types used for diabetes related studies; metabolic pathways involved in blood glucose regulation; metabolite profiles in diabetes; metabolomics biomarkers of diabetes; and limitations and future perspectives.

### 2.6 Data extraction and quality assessment

For each of the study used, we recorded a standardized set of details. These includes author names, year of publication, the title of the study, participants used, analytical methods utilized, biological samples that were tested and the outcomes that were assessed. Also, the methodological quality of the included studies was assessed using a six-point quality assessment framework based on guidelines adapted from the report by Hayden et al. (21). The evaluation encompassed six domains which included the study participation, study design and sample type, metabolomics measurement, outcome assessment, consideration of confounding factors, and suitability of the statistical analysis. Each domain received a point for adequate reporting, with a maximum possible score of six points. Studies that received scores between 0 and 3 were designated as lower quality, whereas those that scored 4–6 were classified as higher quality.

## 3 Results and discussions

### 3.1 Literature search

**Figure 1:**
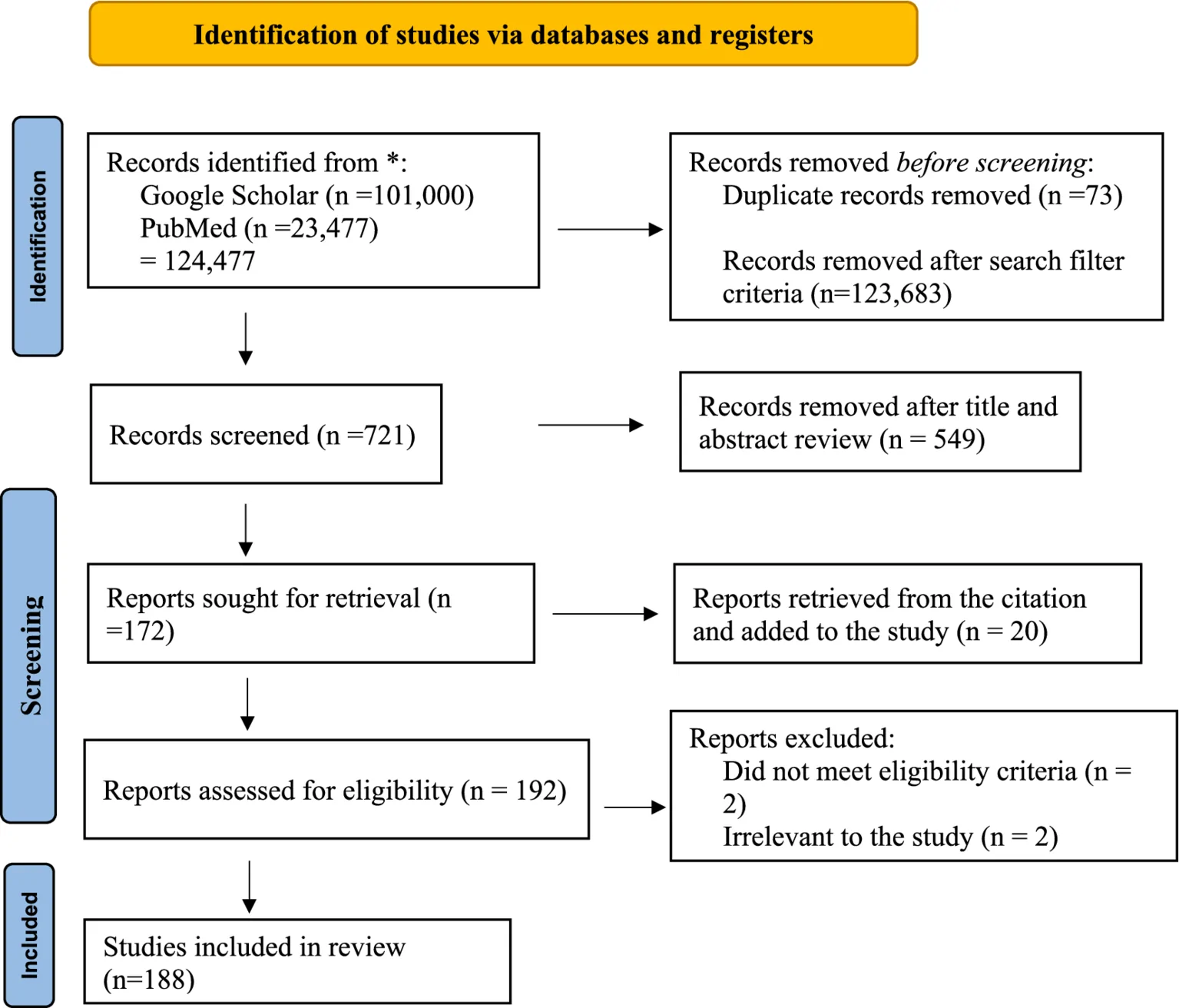
PRISMA flow chart showing data collection and information search for systematic reviews.

The initial literature search yielded a total of 124,477 articles, comprising 101,000 Google Scholar and 23,477 from PubMed. After the advanced search was applied to the filters, the numbers were reduced to 534 and 261 articles, respectively. The search string applied for search on Google scholar which brought the number down to 534, included those published between 2010–2026. The specific key words inputted included “metabolomics, diabetes, blood glucose, biomarkers insulin, NMR, LC-MS, progression, complication, TCA cycle, CE MS, metabolic pathway, targeted and untargeted” and the articles search was also limited to those that were written in English. For PubMed, the criteria remained the same in terms of the year and other filter parameters except for the key words which were inputted. The exact search words inputted on PubMed were “metabolomics (title and abstract) AND diabetes mellitus (title and abstract) AND blood glucose (title and abstract) OR glucose regulation (title and abstract) OR glycemic control (title/abstract)”. This brought the number down to 261 articles. The numbers eliminated were 123,683, bringing it to a total of 795 articles, out of which duplicate articles estimated at 73 were identified and removed, in addition to another 549 excluded after title and abstract review. This gave a total of 172 articles included while about twenty (20) relevant articles were obtained through reference lists and added to the study, making a total of 192 articles. Also, two of the articles did not meet the eligibility criteria while an additional two were irrelevant to the study and both were excluded. In the end, 188 articles were confirmed to be eligible and included in the study. The PRISMA flow diagram (Figure 1) summarizes the literature selection process illustrated.

### 3.2 Overview of the study

A total of 188 articles were included in the study. However, about 89 of the articles addressed diabetes, glycemic control, and insulin resistance, while the remaining 99 articles focused on the technologies used for metabolomic analysis, biomarkers, and metabolites.

## 4 Definition and overview of metabolomics

Metabolomics refers to the complex evaluation of the various metabolites and molecules of low molecular weight (below 1 kDa) in biological systems (22, 23). The field of metabolomics is aimed at capturing the various activities of metabolism which reflect the physiological and pathological state of a biological system over a period (24). By profiling these metabolites, the study of metabolomics provides understanding on cellular metabolism, disease-related changes, and the effects of genetic and environmental changes, which are gaining prominence in the discovery of biomarkers, diagnosis of diseases, and in medicine (25). Analytical procedures, including mass spectrometry (MS) and nuclear magnetic resonance (NMR), are the most common procedures that are employed to quantify and analyze metabolites in a high-throughput manner (26). Metabolomics can be executed in targeted manner, with a focus on already known metabolites, or an untargeted manner, which investigates the numerous molecules in order to reveal several metabolic pathways and new biomarkers (27). As a result of continual technological advancements, metabolomics is gaining more popularity in clinical and research practices, thereby revealing its potentials in diagnosis, medicine, and overall health monitoring at molecular stages.

Metabolomics is important in biological systems through the provision of a complex view of micro molecules that reveal the effective state of biological systems, thereby providing a relationship between genotype and phenotype. Metabolites, which are the main products of gene, transcript and protein interactions, provide a direct narration of cellular processes and influences of the environment, a property that placed metabolomics as an important tool for the understanding of biological networks and their modulations (28). The application of metabolomics in biological systems enables the identification of new biomarkers, understanding of mechanisms of some metabolic diseases such as T2D, and the discovery of drug targets, as well as an understanding on metabolic regulation and adaptation (20, 29). In all, metabolomics enhances biological systems by enabling the transition from descriptive understanding of the biological system to the understanding of the various mechanisms of the biological systems.

Furthermore, the study of metabolomics enables objective analyses of dietary intake as well as analysis of nutrients and different foods and how they impact metabolism and disease progression. The analysis of biofluids such as urine, mucus and blood through metabolomics enhances the identification of specific biomarkers in T2D and other metabolic diseases that reflect the impacts of the consumption of a particular food, dietary patterns, nutrient deficiency, and controlling the limitations of dietary data that are self-reported (30). Thus, metabolomics approaches have led to the advancement and validation of biomarkers by allowing real dietary assessment and improved knowledge of the relationships between diets and health outcomes, including dietary diseases such as T2D, CVD and cancer (31). Metabolomics help promote the advancement of personalized nutritional plans through the revelation of individual responses to nutritional and dietary interventions, thereby promoting precision nutrition (32). The study equally promotes the understanding of the complex relationships between the metabolic processes of various diets and microbiota of the gut and the host, which provide an understanding of the influences of the components of diets on health at the molecular level (33). Considering the challenges faced by the development of metabolomics, such as the need for standardization of methods and the complex nature of reading the data, its integration into nutritional research is creating impacts in the field of nutritional sciences and holds great potential for evidence-based guidelines for diets and personalized nutritional recommendations (34).

Due to the ability of metabolomics to provide detailed analysis of the rate of metabolisms of individuals, it is central to personalized nutrition and medicine, which reflects genetic and environmental influences. The profiling of metabolites in biological samples through metabolomics study enables the identification of biomarkers of T2D that are capable of disease diagnosis, predict disease vulnerability, and monitor therapeutic responses with high sensitivity and specificity (35). This approach promotes the stratification of patients by enabling person-centered treatments according to the unique metabolic profile of the individual and how they respond to medications or their susceptibility to disease, a popular concept known as pharmacometabolomic (36). Metabolomics have been considered as a valuable tool for the monitoring of disease progression and effectiveness of treatments in real time, which is vital for the optimization of patient care (36).

### 4.1 Analytical platforms used in metabolomics

To comprehensively profile some micro molecules, metabolomics basically relies on several analytical platforms with each providing unique strengths. NMR spectroscopy, liquid chromatography mass spectrometry (LC-MS), gas chromatography mass spectrometry (GC-MS) and capillary electrophoresis mass spectrometry (CE-MS) are recognized for their reproducibility, accuracy, and ability to clearly identify metabolites that are unknown, and its non-destructive nature and unambiguous sample preparation demands as reported on Table 1. However, some methods showed less sensibility compared to others (37). These platforms are mostly combined during metabolomics analysis since no single method or platform can capture the entire metabolome as well as to also ensure reliability.

**Table 1:** Some metabolomics-based studies and their application in diabetes and related metabolic diseases.

S/N	Study	Participants	Metabolomic technique	Samples assayed	Outcome	References
(A) Human observational studies						
1	T2D prediction and progression	503 T2D case-control pairs	Untargeted metabolomics (LC-MS)	Fasting plasma	46 predictive metabolites were identified and reported in the study to be associated with beta cell dysfunction and T2D progression	(175)
2	Early prediction of gestational diabetes	Pregnant women in their early stage	UHPLC-MS/MS (untargeted)	Plasma	Altered early pregnancy thyroid markers and lipid species were associated with the development and progression of GDM. Risks were identified with high accuracy before clinical diagnosis	(176)
3	Gut microbiota pattern and prediction of DKD	Human patients with and without DKD	LC-MS and NMR spectroscopy	Serum and fecal sample	Serum metabolomics combined with gut microbiota when profiled can be used as a strategy to predict DKD. Predictors of renal function decline in T2D were also discovered in the study.	(177)
4	Plasma metabolite biomarkers of diabetic nephropathy in an untargeted metabolomics	Patients who had diabetic nephropathy and those without nephropathy as well as healthy controls	Untargeted metabolomics (LC- MS)	Plasma	Amino acid, lipids and energy metabolism pathways in the plasma that were altered were identified and helped distinguish between T2DM patients without nephropathy and healthy controls. Potential biomarkers for early diabetic nephropathy detection were proposed	(178)
5	Profiling metabolically health morbid and morbid obesity with associated T2D using Lipidomic	209 women in total were used as participants comprising of control as well as morbidly and healthy morbid obese women associated with T2D	LC-MS	Serum	Morbid obese women exhibited increased levels of ceramide, sphingomyelin, triglycerol and fatty acids and a reduced acylcarnitine, bile acid, phosphatidylcholines compared to normal weight women. Morbid obese women showed elevated triacylglycerols, phosphatidylcholine, phosphoethanolamine than healthy morbid obese women	(179)
(B) Human interventional and clinical studies						
6	Metabolomic fingerprints of medical therapy versus bariatric surgery in patients with T2D and obesity	Adults with T2D and obesity randomly assigned to medical therapy, roux-een-Y gastric bypass or sleeve gastrectomy	Untargeted metabolomics using UPLC-MS/MS, combined with machine learning and linear mixed-effect and modelling	Plasma samples that were taken at baseline and 24 months after intervention.	Bariatric surgery produced greater remodeling of plasma metabolome compared with medical therapy. Both surgical procedures increased lipid and amino acid related metabolic signatures related to medication use. 2-hydroxydecanoate was identified as one of the most distinguishing metabolites with its change correlated significantly to reduction in fasting glucose	(180)
7	Dapagliflozin modulation OF plasma lipidomic profile and urinary metabolite excretion in T2D	Patients with T2D and hypertension randomized to dapagliflozin and hydrochlorothiazide	Plasma lipidomic and metabolomics using high resolution MS	Fasting plasma and urine	In the study, hydrochlorothiazide when compared to dapagliflozin increases plasma metabolites (isoleucine, citrate, and beta-hydroxybutyrate), there was a decrease in lactate. Free fatty acids, sphingomyelins and lysphosphatidylcholines were also increased. In the urine, a major metabolic adaptation where in amino acids, lactate, TCA cycle metabolites and electrolyte also increased	(181)
8	A CORDIOPREV Trial (NCT00924937)	Study was made 1,002 participants with established cases of T2D or coronary heart disease.	MS based profiling (Targeted)	Fasting and OGTT serum samples	12 metabolites which included amino acids and lipids were identified and predictive of T2D remission following a long term Mediterranean dietary intervention	(182)
9	Diabetes remission clinical trial	The study was made of 298 patients with T2D randomized to weight management routine	LC-MS (untargeted) and H-NMR (targeted)	Fasted serum sample	There was reversal of diabetic hall marks from the study. It included sharp reductions in BCAAs, sugars and LDL triglycerides	(183)
10	A randomized clinical trial of BCAA catabolism in patients with T2D	Patients randomized into placebo and intervention group	LC-MS	Plasma	The study showed metabolic effects of pharmacologic activation of BCAA catabolism in T2D	(184)
11	Effect of empagliflozin on plasma lipid metabolome in patients with T2D	T2D patients	GC-MS	Serum	An Increase in omega 3 related metabolite, unsaturated fatty acids and ketone related metabolites and decreased saturated fatty acid, some amino acid metabolites linked to insulin resistance were the key highlights from the study	(185)
(C) Experimental animal studies						
12	Hepatotoxicity of Gynura segetum in diabetes models	Rat (clinical proxy)	GC-MS	Serum	The study identified 26 differential metabolites involved in phenylalanine and glyoxylic acid metabolism, which provides biomarkers for liver injury in metabolic syndrome	(186)
13	Plasma metabolomic profiling of glucose tolerance	Nile rat models of spontaneous T2D	UHPLC-MS/MS (untargeted)	plasma	The study identified metabolic signatures that distinguish healthy animals from prediabetic and diabetic animals. Of these, bile acids and lipid metabolism exhibited the most significant alterations.	(187)
14	Lipid metabolism dysregulation in mouse models with DM	Diabetics induced mouse models	LC-MS based lipidomic + MS/MS	Brain, serum, liver, kidney, heart tissues	The study showed a relationship between T2D, AD, and hypertension stating their common metabolic pathways. It confirmed that there was an increased risk of Alzheimer in T2D patients	(188)

#### 4.1.1 Nuclear magnetic resonance (NMR)

NMR spectroscopy is an analytical platform that is foundational in metabolomics studies. It relies on the principle that several nuclei of atoms are capable of resonating at some characteristic frequencies when exposed to radiofrequency pulses in a nuclear field, thus revealing details of the molecular structure and environment (38). In metabolomics studies, NMR is applied widely in the profiling of metabolites in biofluids, tissues, and some intact organisms to detect onset and progression of diabetes. This supports its application in the diagnosis of disease biomarkers such as T2D, dietary or nutritional studies, environmental monitoring and biological studies (39). Its advantages include non-destructive analysis, requiring less cumbersome sample preparation and high reproducibility. It also possesses the ability to reveal both qualitative and quantitative details simultaneously, making it an important analytical platform for large-scale and longitudinal studies (40). NMR can clearly identify unknown metabolites as well as trace the metabolic pathways such as glucose metabolism and onset of T2D with the aid of isotope-labeled substrate, which offer clear understanding of their metabolic fluxes (41). Despite these advantages of the NMR platform, it is faced with some limitations, which include low sensitivity compared to mass spectrometry, thereby limiting identification to metabolites that are relatively abundant and demanding a large volume of samples. The high cost of instrumentation and the need for specialized expertise in the interpretation of the spectra pose extra challenges in the use of NMR (42). Recent technological advancements on NMR such as the use of higher magnetic fields and enhanced probe technology, as well as automation, are improving the sensitivity and throughput of NMR, which increases the utilization of NMR in metabolomic studies especially in glucose metabolism and T2D (43).

#### 4.1.2 Liquid chromatography-mass spectroscopy (LC-MS)

One of the leading analytical platforms in metabolomics is LC-MS, which combines its ability to separate liquid with the sensitive detection and identification power of mass spectrometry. The principle is based on the separation of complex mixtures of metabolites according to their chemical properties with the aid of LC, followed by detection and quantification of the metabolites through the measurement of mass-to-charge ratio of the MS (44). The analytical platform is mainly used in targeted and untargeted metabolomics for the discovery of biomarkers of T2D, nutritional studies, development of drugs, and disease diagnosis, because it can analyze a wide range of metabolites, such as urine, blood, and tissues (45). The major advantages of LC-MS in analyzing glucose metabolism for T2D include high sensitivity, broad coverage of metabolites, efficient analysis, and versatility, thereby rendering it the most popular platform in many other metabolomic studies (46). The LC-MS platform is capable of detecting metabolites that are low and can be employed for different types of samples such as the plasma and urine sample for T2D that will meet certain research objectives and questions (47). Despite these advantages of LC-MS, there are several limitations to its use for metabolomic analysis in T2D and other metabolic diseases. These includes difficulty in identification of compounds (with many identifications features remaining unannotated), poor and incomplete coverage of the metabolome as a result of diverse properties of metabolites, and the need for thorough preparation of samples to mitigate against the effect of the matrix and ensure reproducibility (48). The complex nature of the LC-MS platforms demands expertise, and the analysis of the data could be cumbersome (46).

#### 4.1.3 Gas chromatography-mass spectrometry (GC-MS)

Another key analytical platform for metabolomic studies, such as that of T2D, is GC-MS. The GC-MS operates first by the separation of thermally stable and volatile compounds through gas chromatography (GC), followed by identification and quantification of these compounds according to their mass-to-charge ratios through MS (49). This platform is proficient in profiling of micro molecules, such as amino acids, fatty acids, sugars, and organic acids, particularly after the modification (derivatization) of the compounds to enable a stable and volatile component (50). The major strength of GC-MS relies on its high sensitivity, robust reproducibility, and its ability to quantify metabolites for the detection of T2D and other diseases, a feature that is supported by extensive spectral data that will enhance a reliable identification of compounds or metabolites in the blood or urine. The GC-MS is basically employed for untargeted metabolomics, the discovery of biomarkers in T2D, and the study of other primary metabolites in several biological and environmental samples.

Meanwhile, some of the challenges of the use of GC-MS include the derivatization of metabolites that are non-volatile, a phenomenon that can lead to the complication of sample preparation and introduce variability, and its inability to analyze large and thermally stable compounds (51). Few metabolites can be analyzed through GC-MS compared to LC-MS, and its data analysis can be more cumbersome because of its complex disintegration pattern. However, there are recent technological advancements of GC-MS that have been developed to improve metabolite coverage, sensitivity, and quality of data, a feature that is conferring GC-MS as more powerful and suitable for the study of T2D and many other metabolic diseases (52).

#### 4.1.4 Capillary electrophoresis-mass spectrometry (CE-MS)

CE-MS is a well-developed and important platform for metabolomics analysis of metabolites for the assessment of biomarkers for T2D, especially its ability to effectively separate and detect high polar and charged metabolites that are usually difficult for other analytical platforms and techniques (53). The operating principles of CE-MS include employing an electric field to separate metabolites in a capillary according to charge-size ratio, accompanied by MS detection for sensitive identification and quantification (54). It is mainly applied in biomedical sciences, clinical diagnosis of metabolic diseases such as T2D, microbial analysis, plant, and food metabolomics. It is usually preferred for the analysis of samples of small sizes and volumes, or volume restricted samples, including cerebrospinal fluids or single cells (54).

The major advantages of CE-MS include high efficiency in separation, the ability to minimize the sample and reagents, and the ability to profile a wide range of ionic and polar metabolites that cannot be easily assessed with the use of LC-MS and GC-MS (55). Furthermore, the CE-MS platform provides robust quantification of metabolites for diagnosing and monitoring T2D. Recent advances in interfacing technology and standards and protocols have showed great reproducibility and reliability in most analyses (56). Meanwhile, the drawbacks of the use of CE-MS include technical hitches in the development of methods, lower sensitivity compared to the LC-MS platform, and differences in migration times, which can lead to complications of metabolite identification across laboratory comparison (57). Recent developments and approaches of CE-MS analysis, such as the use of appropriate and effective electrophoretic mobility or more reproducible compound analysis, are addressing these challenges of expanding the application of CE-MS in metabolomic analysis (58). The summary of the various metabolomics platforms, their principles, strengths, and challenges are shown in Table 2.

**Table 2:** Metabolomic analytical platforms for the detection and assessment of diabetes biomarkers.

Platform	Operating principle	Advantages	Challenges	Applications
Nuclear magnetic resonance (NMR) spectroscopy	Employs strong magnetic fields and radio waves to detect nuclei in metabolites excitation	High reproducibility with minimal chemical variations between runs Can quantify metabolites using a single reference standard (no external standard required) Nondestructive Sample preparation not required	High capital and maintenance cost Poor sensitivity Limited metabolite coverage when compared with MS based platforms	Analysis of structures in biofluids like blood, urine and serum Quantitative profiling of abundant metabolites in biofluids Metabolic monitoring in diabetes study
Liquid chromatography-mass spectrometry (LC-MS)	Separates metabolites based on chromatographic retention, accompanied by detection based on mass-charge ratio	Broad coverage of metabolite High analytical sensitivity Suitable for thermolabile and nonvolatile compounds Compatible with untargeted workflow	Sample preparation maybe cumbersome leading to possible ion suppression	Suitable for targeted and untargeted metabolomics, studies of drug metabolism Useful in pharmacometabolomic and lipidomic study
Gas chromatography-mass spectrometry (GC-MS)	Gas chromatography separates volatile compounds while the identification of metabolites and other compounds are achieved by mass spectral detection	High reproducibility due to standardized electron ionization spectra High chromatographic resolution Have a comprehensive spectral library which allows for confident metabolite identification	Requires modification Can only be used for volatile or chemically derivatized compounds or metabolites	Evaluation of organic acids, fatty acids, amino acids as well as some environmental and biological metabolites Assessment of energy metabolism alteration in diabetes
Capillary electrophoresis-mass spectrometry (CE-MS)	Employs electric fields in the separation of ions through charge and size, followed by detection and identification through mass spectroscopy	Highly effective for ionic and polar compounds Does not require much sample volume	Have limited reproducibility, less sensitive compared to LC-MS	Applied in profiling of metabolites of ionic species Assessment of metabolic pathways related to insulin resistance and glycolysis For profiling of charged peptides and amino acids

Source: Hajnajafi and Iqbal (173) and Aderemi et al. (174).

### 4.2 Metabolomics strategies

#### 4.2.1 Targeted and untargeted metabolomics

Metabolomics is a global method of profiling, employed comparatively to detect, quantify, and discover metabolites in samples. The major objective of this method of analysis is to identify metabolite perturbations that is influenced by certain conditions such as diseases and genetics (59). As a result of these biochemical processes in tissues or cells, metabolomics is an important analytical tool that will ensure proper comprehension of the changes in metabolism, genes and protein pathways that modulate the level of metabolite *in vivo* (60). Metabolomics are generally grouped into 2 broad groups, namely, targeted, and untargeted metabolomics.

Targeted metabolomics includes those analytical platforms that are aimed at quantifying predetermined groups of chemically or biochemically analyzed metabolites, usually preselected due to their relevance to specific state of disease or biological pathways (61). This type of metabolomics analysis employs standard techniques to enhance sensitivity, reproducibility, and quantitative accuracy for some preselected metabolites, such as fatty acids, amino acids, and other micro molecules (62). This method of analysis has gained relevance in clinical diagnosis, discovery of biomarkers, subtyping, and analysis of glucose metabolic pathways with respect to DM. This is because it is more precise in the analysis of metabolites quantification, reproducibility, and ability to compare results across laboratories, particularly when standard protocols and reference materials are employed (63). In addition, this type of metabolomics analysis is generally limited to few ranges of metabolites in comparison with the untargeted methods, with chances of missing novel or unintended compounds (64).

Meanwhile, untargeted metabolomics aims at analyzing the levels of all metabolites in samples, such as metabolites with structures that are yet to be annotated. This method of analysis is focused on the capturing of a broad spectrum of both preselected and unknown metabolites, ensuring the discovery of novel metabolic pathways, disease mechanisms, and biomarkers of metabolic diseases such as T2D (65). Untargeted metabolomics is mainly employed in biomedical, environmental, plant and microbial evaluations to compare metabolic profiles that are influenced by several conditions (66). Also, untargeted form of metabolomics analysis tends to be unbiased and have the potential to reveal unknown metabolic modifications. It also presents vital challenges, such as complex data processing, difficulties of metabolite identifications, and the presence of many unknown or redundant signals (46). The major bottleneck for this method is identification, because many signals do not match existing databases, usually as a result of informatic artifacts or contaminations by chemicals rather than novel compounds (67).

## 5 Biological sample types used for diabetes related studies

Various samples such as the tissues, blood (plasma and serum), urine, and saliva are bio fluids employed for metabolomics analysis. The human biofluids are endogenous bodily fluids secreted naturally by the human body, usually assessed in metabolomics analysis for T2D especially the urine and plasma. The presence of certain urinary markers, such as albumin and sodium excretion are essential in the identification of T2D and its associated microvascular complications (68). These renal tubular biomarkers have been shown to have significant potential for early detection and monitoring of renal injury in diabetes, thereby allowing timely intervention to protect kidney function and improve patient conditions. MicroRNAs, like microRNA 126 and microRNA 770, are indicators for diabetic nephropathy (69). They are present in the blood, tissue, saliva, and urine and are mostly bound to proteins. MicroRNA 126, basically expressed in endothelial cells, is strongly linked to vascular morbidity in diabetic nephropathy, while microRNA 770, which modulates gene expression in cell growth and metabolic diseases, may serve as a remedial way for managing the disease (70). These two microRNAs exhibit considerable ability as noninvasive biomarkers, with the prospect of facilitating timely diagnosis and continuous assessment of diabetic nephropathy. Combining urinary biomarkers with sophisticated diagnostic equipment, such as continuous glucose monitoring (CGM) and wearable health devices, could improve diabetes management by providing a more extensive evaluation of the disease progression (71).

At present, the current methods for diagnosing diabetes basically entails the assessment of fasting blood glucose level, oral glucose tolerance test, and evaluation of glycosylated hemoglobin (72). Also, with advancement in technologies, methods such as MS and NMR are especially important in the detection and management of T2D and holds a great potential for the future (73). The science of metabolomics involves the investigation and analysis of low molecular weight compounds (metabolites) usually less than 1,000 Da with a primary focus on their collection, measurement, and subsequent interpretation (74). These metabolites include lipids, amino acids, nucleotide, lipids. In many biological organisms, they act as important biomarkers that modulates various metabolic processes (75). Metabolites have been found to play a critical role in pathophysiological responses in the human body. In metabolomics, highly sensitive analytical techniques are deployed to uncover normal and abnormal perturbations and mechanisms of the body through detection of minute biological changes (76). Consequently, the development of a disease can be diagnosed by observing the participation of these metabolites in various reactions by interactions with various metabolic pathways and different metabolites that are produced in the blood, urine, or saliva.

**Figure 2:**
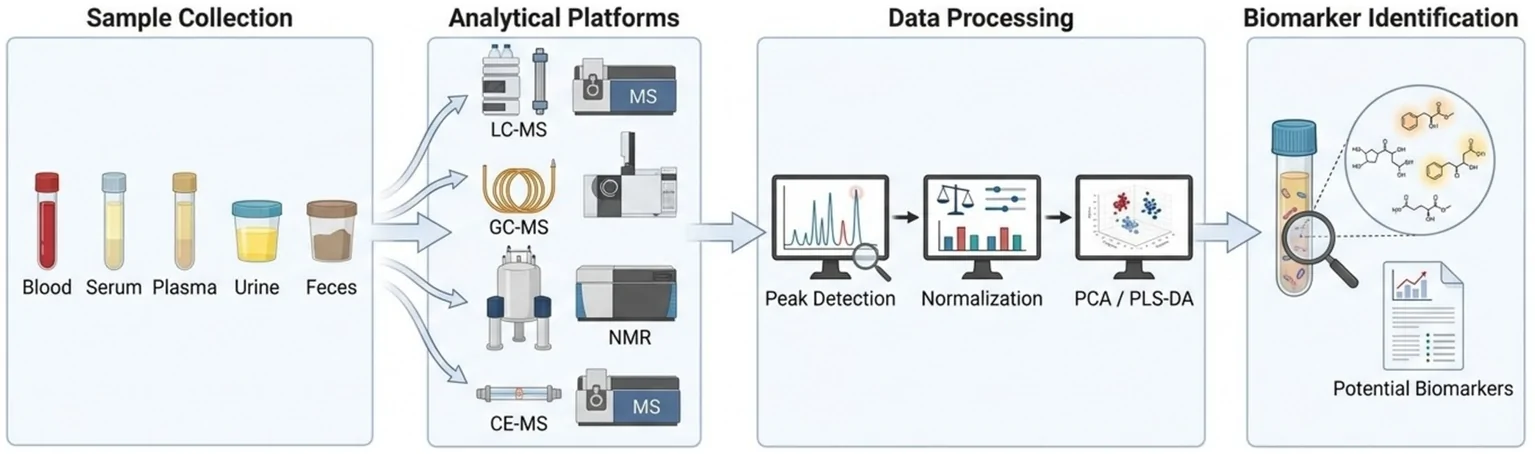
The basic principle underlying the use of metabolomics [adapted from: Ren et al. (167), Huang et al. (168), and Long et al. (169)].

Currently, NMR spectroscopy, LC-MS, and GC-MS are the most commonly used methods to detect metabolites for T2D. The NMR is usually simpler to use and does not need much sample preparation, making it more user friendly. On the other hand, MS is more responsive than NMR and is especially useful in the detection of metabolic markers in heterogeneous biological samples (77). The basic principle underlying the use of metabolomics is shown in Figure 2.

In patients diagnosed with T2D, the concentrations of branched-chain amino acids have been observed to reach up to 1.5-fold or even 2-fold higher levels compared to those observed in healthy subjects. However, the mean change remains below 1.5-fold (1.2 to 1.3) (78). It is established that increased concentration of branched-chain amino acids in the blood is an excellent marker of impending insulin resistance among T2D patients (79). Also, Damanhouri et al. (80) pointed out that there were elevated plasma concentrations of branched-chain and aromatic amino acids, along with increased glutamate-to-glutamine levels, in diabetic individuals compared to healthy subjects. As such, a substantial body of research has demonstrated that there exists a significant correlation between T2D and amino acids and this has significant implications for the management of T2D. Similarly, individuals with elevated concentrations of fatty acids and low carbon lipids, like glycerophospholipids and sphingomyelins, have been diagnosed with T2D (81). Sugar metabolites, such as glucose, dihexose, mannose, arabinose, and fructose are reported to show a positive correlation with T2D (82).

## 6 Metabolic pathways involved in blood glucose regulation

Blood glucose regulation involves a variety of related metabolic pathways that help regulate the blood through balancing the rate of glucose production, usage, and storage. The process known as glucose metabolism is among the central pathways that are responsible for homeostasis, and these include glycolysis and glycogenesis.

Blood glucose homeostasis requires precise coordination between glucose utilization and endogenous production (83). During glycolysis, glucose is broken down to pyruvate, generating ATP and other metabolic intermediates required for energy generation (84), and its dysregulation through insulin resistance or impaired hepatic and β-cell enzyme activity directly disturbs glucose utilization in diabetes (85). Insulin drives glycolytic flux by upregulating glucokinase, PFK-1, and pyruvate kinase and by raising fructose 2,6-bisphosphate; glucagon and catecholamines reverse this in the fasted state (86, 87). This hormonal control is reflected directly in the diabetic metabolome, where elevated circulating lactate, pyruvate, and the glycolysis-linked metabolite, α-hydroxybutyrate, are among the earliest detectable markers of insulin resistance and impaired glucose tolerance (88, 89).

**Figure 3:**
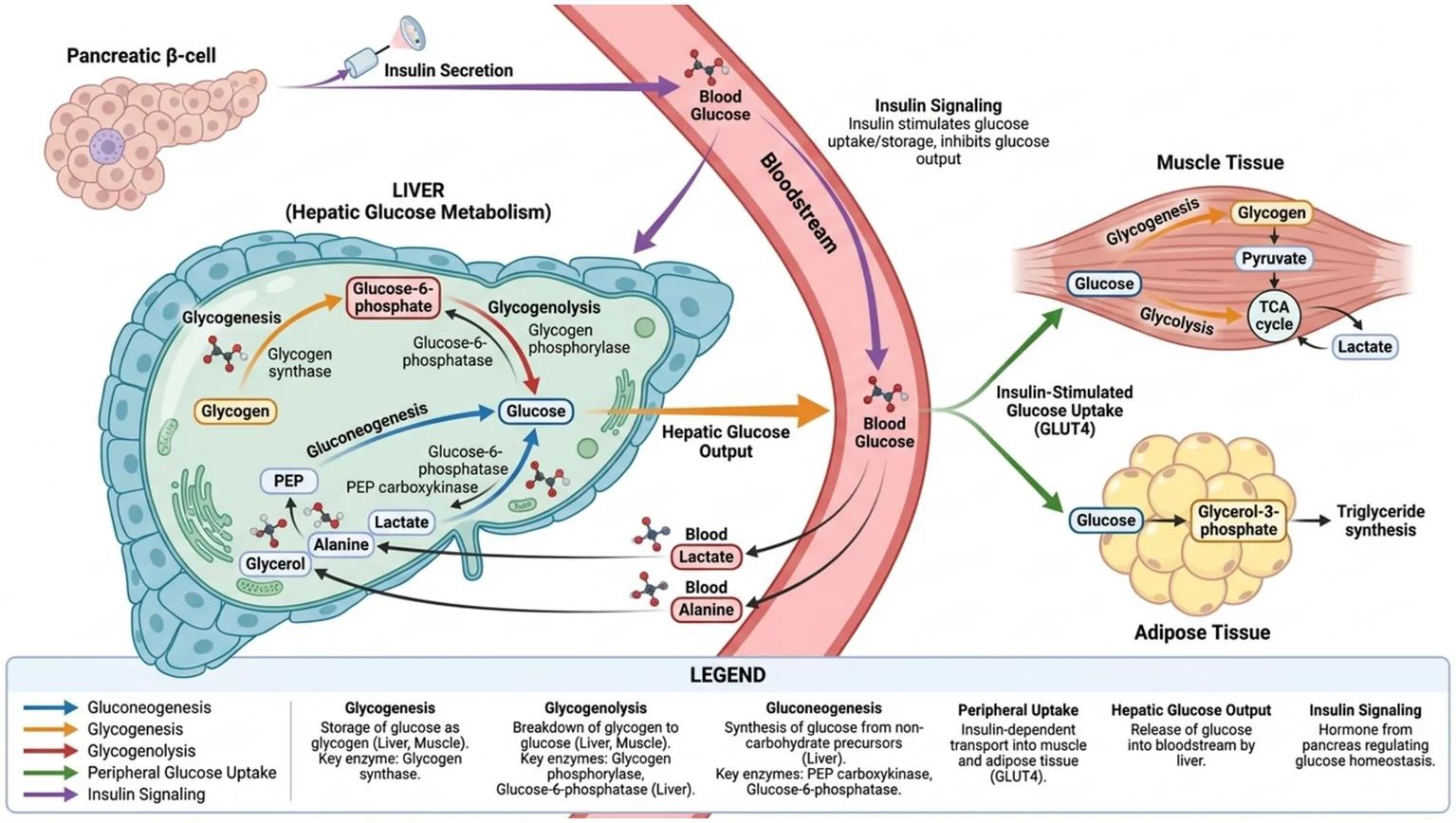
Metabolism of glucose in the major metabolic organs and insulin regulated pathways [adapted from: Samuel and Shulman (170), Magkos et al. (171), and An et al. (172)].

Gluconeogenesis generates glucose from lactate, glycerol, and amino acids and is essential during fasting, exercise, or stress, with the liver and, to a lesser extent, the kidney as primary sites (90, 91). Insulin suppresses gluconeogenic gene expression (PEPCK, G6Pase, PCK1) via Akt signaling, while glucagon and cortisol induce it through cAMP-CREB signaling (92–94). Failure of insulin to suppress hepatic gluconeogenesis is a defining feature of T2D and drives fasting hyperglycemia (95, 96). Metabolomic studies have been shown to directly capture this defect. Gluconeogenic amino acids, including the branched-chain amino acids leucine, isoleucine, and valine, as well as the aromatic amino acids phenylalanine and tyrosine, have been found to be elevated years before diagnosis. These amino acids have been demonstrated to predict future T2D risk more strongly than fasting glucose alone (97). A summary of glucose metabolism, including these diabetes-associated metabolite shifts, is shown in Figure 3.

Downstream of glycolysis, the TCA cycle oxidizes acetyl-CoA from carbohydrates, fats, and proteins. This generates NADH and FADH_2_ that feed oxidative phosphorylation to produce cellular ATP, while also supplying intermediates for amino acid, lipid, and nucleotide biosynthesis (98, 99). Because the cycle sits at the intersection of all major fuel sources, disruption of TCA/oxidative-phosphorylation coupling is implicated in insulin resistance and diabetic complications (100). Metabolomics profiling of diabetic plasma and urine has been shown to consistently exhibit altered TCA intermediates. Specifically, citrate and succinate levels increase, while α-ketoglutarate and other intermediary compounds decrease in comparison to non-diabetic controls. This pattern has been attributed to impaired anaplerotic flux and incomplete fatty acid oxidation in the insulin-resistant state (101). Furthermore, anaplerotic reactions renew the intermediates of the TCA cycle so as to sustain the production of energy and biosynthetic requirement (102). The TCA cycle and oxidative phosphorylation are crucial for cellular energy, flexible metabolism, and adaptation to pathological and physiological challenges.

Again, the parallel branch of glucose metabolism, which is the pentose phosphate pathway (PPP), generates NADPH and ribose-5-phosphate. The NADPH helps to maintain redox balance and supports fatty acid, cholesterol, and nucleotide synthesis, while ribose-5-phosphate supplies nucleotide production (103, 104). Because oxidative stress is central to T2D pathophysiology, reduced PPP flux limits the regeneration of reduced glutathione. This compounds oxidative damage in insulin-target tissues, while G6PD deficiency illustrates the cost of impaired redox defense (104). Alterations in the ribose-5-phosphate and NADPH/NADP+ ratios serve as indicators of the oxidative stress that accompanies and may occur prior to the development of hyperglycemia. Furthermore, the PPP flux is increased in both T2D and cancer to meet elevated demands for NADPH and nucleotides (104, 105).

Beyond central carbon metabolism, lipid metabolism, including its storage, breakdown, and signaling functions of triglycerides, fatty acids, phospholipids, and cholesterol is tightly coupled to insulin and glucagon signaling (106). Insulin promotes triglyceride storage and suppresses lipolysis while glucagon reverses this during fasting. In insulin resistance, suppression of lipolysis fails and this drives excess free fatty acid release and contributes to hepatic steatosis and hyperglycemia (107, 108). The major focus of diabetes lipidomics usually entails the investigation of lipolytic dysregulation, in conjunction with fluctuations in particular lipid species (109, 110). Also, elevated levels of diacylglycerols and sphingomyelins, along with modified phospholipid and triglyceride subclasses, serve as distinguishing characteristics between diabetic and healthy profiles (111, 112). These molecular signatures have the capacity to serve as predictive indicators of disease risk. Membrane glycerophospholipid remodeling directly affects insulin signaling. Altered phosphatidylinositol pools disrupt GLUT4 trafficking and β-cell insulin secretion, and remodeled glycerophospholipid profiles in skeletal muscle are linked to insulin resistance in obesity (113–115).

Ceramides are the most consistently implicated lipid mediators of insulin resistance in diabetes metabolomics. Their accumulation in liver and skeletal muscle impairs insulin signaling and is a core lipotoxic mechanism in T2D pathogenesis, driven largely by saturated fatty acid flux into ceramide synthesis (116). Circulating ceramides are similarly implicated in obesity-related insulin resistance, with hypoxia proposed as a contributing mechanism (117, 118). Experimentally, lowering ceramide levels helps to improve insulin signaling and glucose metabolism in diet-induced obese mice, underscoring their value as circulating biomarkers of hepatic insulin resistance.

### 6.1 Metabolic alterations in prediabetes

Prediabetes, also known as impaired glucose tolerance (IGT) or impaired fasting glucose (IFG), is characterized by elevated glucose levels in the blood that are not up to the diagnostic criteria for T2D, and this impaired glucose homeostasis is an early indicator of developing T2D (119). Insulin resistance and decreased β-cell function cause fluctuations in plasma glucose levels, leading to pre-diabetes and eventually T2D (120).

In the prediabetic state, the metabolic profile undergoes an early shift that reflects subsequent development of insulin resistance. A substantial body of research has demonstrated that large cohort metabolomics studies consistently show elevated circulating branched-chain amino acids such as leucine, isoleucine, and valine, increased aromatic amino acids like phenylalanine and tyrosine, and higher levels of lipid intermediates, such as acylcarnitine and diacylglycerols (121, 122). This is associated with reduced glycine and other insulin-sensitizing metabolites. These alterations are not incidental but constitute a reproducible metabolic signature that tracks with impaired glucose regulation and often manifests prior to the development of T2D (123). Consequently, they serve as useful biochemical indicators of prediabetic progression.

Though prediabetes can lead to diabetes, the risk varies depending on the definition that was utilized (119, 124). At the metabolite level, large human cohort metabolomics studies have consistently demonstrated that prediabetes is associated with gradual elevated circulating acylcarnitine and this reflects impaired fatty acid oxidation (125). Also, higher presence of lactate indicates altered glycolytic flux, and disturbances in lipid metabolism, which includes glycerophospholipids and sphingolipids such as ceramides (126). These changes tend to mark the early stage of mitochondrial dysfunction and insulin resistance and they tend to intensify and become more dysregulated as individuals progress toward overt T2D (127). It is mostly characterized by a fasting glucose level between 100 and 125 mg/dL, a glucose level of 140 to 199 mg/dL assessed two hours following a 75-g oral glucose load (2-h glucose), or a glycated hemoglobin level (HbA1C) of 5.7 to 6.4% or 6.0 to 6.4% (120, 128, 129). People with abnormally elevated fasting glucose, 2-h glucose, or HbA1C levels in the prediabetic range are more likely to develop T2D (124, 130). However, not all patients who have prediabetes proceed to DM.

### 6.2 Metabolic alterations in type-1 diabetes (T1D)

T1D, also known as autoimmune or insulin-dependent diabetes, is an autoimmune disorder characterized by the targeted breakdown of β-cells in the islets of Langerhans, which are responsible for producing insulin (131). This causes a deficiency of insulin, making people with T1D require insulin for the rest of their lives (131). This condition can be caused by a combination of several predisposing factors that affect the homeostatic balance of blood glucose, negatively impacting the relationship between insulin-production efficiency and immunological reactions, which varies by age (132).

In T1D, the metabolic signature is most pronounced in plasma, with corresponding changes in the urine that reflect absolute insulin deficiency rather than insulin resistance. Plasma metabolomics consistently reveals elevated levels of ketone bodies, such as β-hydroxybutyrate and acetoacetate, as well as increased levels of acylcarnitine derived from free fatty acids (133). Additionally, there is a notable rise in amino acid catabolism, particularly that of branched-chain amino acids especially during energy shortage (134). These alterations indicate a notable shift toward lipid oxidation and ketogenesis (135). Glucosuria which occurs when the renal threshold of glucose is exceeded in the urine as well as ketonuria, and increased excretion of organic acids are associated with impaired glucose utilization and acid base imbalance for people with T1D (136). Collectively, these plasma and urine signatures indicate uncontrolled substrate accumulation due to the absence of insulin leading to incidences of T1D.

In the study by Cai et al. (112), patients with T1D who were under glycemic control exhibited a general reduction in plasma lipid species, particularly TAGs, DAGs, phosphatidylcholines (PCs), and phosphatidylethanolamines (PEs). This indicates that these lipid classes are altered from metabolic biomarkers of the disease.

Another indicator of T1D is the disorder of the exocrine pancreas, leading to a decrease in its size, weight, and volume, as well as a reduced level of enzymatic serum in the enzymes of the exocrine pancreas (137). Reduced pancreatic mass and blood serum trypsinogen levels correlate with the onset of the illness (138). Studies have shown about 25 to 30 percent loss of pancreatic volume in early cases of T1D and up to 50% loss in patients who have had the disease for years, when compared with non-diabetic patients (139, 140).

### 6.3 Metabolic alterations in type-2 diabetes (T2D)

Type-2 diabetes (T2D) manifests as elevated blood glucose and severe insulin resistance, affecting the liver, fatty tissues, and muscle cells in the skeletal system. It has a diverse etiology, driven by genetic and environmental factors (141). Usually, insulin functions mainly to enhance glucose absorption in adipose and muscle cells while restricting the synthesis of glucose from the liver. Therefore, insulin signals play a crucial role in insulin activity throughout various tissues, including the liver, muscle, and adipose tissue, as well as the pancreas’ β-cells, brain, the endothelium of blood vessels, and generally maintain homeostatic metabolic processes (142, 143).

Furthermore, disturbances in metabolism manifest over time and are detected earlier in plasma, with urine changes becoming more apparent in advanced or poorly managed cases. Plasma profiles, just like that of T1D, are characterized by persistent elevation of branched-chain amino acids (leucine, isoleucine, and valine), increased aromatic amino acids (phenylalanine and tyrosine), accumulation of short- and medium-chain acylcarnitine, and dysregulation of lipid metabolism, including triglycerides, diacylglycerols, and sphingolipids (135). These alterations are indicative of progressive insulin resistance, mitochondrial overload, and impaired fatty acid oxidation. Again, alterations in TCA cycle intermediates, and shifts in microbial-derived organic acids may be detected in urine; however, these are less consistent than plasma lipid and amino acid signatures. In essence, T2D is characterized by an ongoing imbalance in the processing of lipids and amino acids, as opposed to the occurrence of acute ketone-driven energy failure.

## 7 Early biomarkers of insulin resistance (IR)

Insulin resistance (IR) has been identified as a primary genetic triggering pathway to T2D, predating the start of the disease (144, 145). Though the use of “hyperinsulinemic-euglycemic glucose clamp” is currently acknowledged as the benchmark technique or standard for detecting IR, it is too laborious and burdensome; thus, there is a need for a scientifically vetted, affordable, and efficient diagnostic method (146). This need has spurred the research community in placing high priorities on the establishment of vetted, and dependable IR biomarkers (144, 146).

One of the key biomarkers of IR is elevated insulin levels in the blood. In order to maintain a normal glucose level in the body, careful coordination of insulin response, β-cell activity, and insulin elimination processes is expedient. Disrupting this relationship, basically because of insulin resistance, disrupts the optimum balance across insulin and blood glucose levels in the baseline, leading to increased or excessive insulin production to normalize the blood glucose level in the body (147, 148).

Non-coding ribonucleic acid (RNAs) are emerging as another potentially useful biomarker and treatment targets for insulin resistance, especially owing to how they regulate certain functions in the body (149–151). The livers of patients with IR have been found to exhibit dysregulated microRNA and long non-coding RNA function, as many RNA transcripts regulate liver-specific insulin-mediated pathways. One common symptom observed before T2D diagnosis is a dysfunctional metabolic process in the liver, which, in turn, results in IR and an imbalance in blood glucose levels. Thus, T2D occurrence can be minimized significantly when methods are put in place to diagnose and treat liver IR early (149). de Klerk et al. (150) also conducted a clinical study on 412 diabetic individuals in the Hoorn Diabetic Care System, who were divided into five groups based on their age, BMI, HbA1c, C-peptide, and HDL cholesterol, to evaluate the disparity in their small non-coding RNAs. The five groups include severe “insulin-deficient diabetes (SIDD), severe-insulin resistant diabetes (SIRD), mild obesity-related diabetes (MOD), mild diabetes (MD), and mild diabetes with high HDL cholesterol (MDH)”. The result shows that the group characterized by IR had anomalous sncRNA expressions, whereas the extreme BMI group exhibited eight differential sncRNA expressions (150, 151).

Other biomarkers implicated in the early detection of IR are the expression of some genes, such as the glucokinase (GCK), fructose-1,6-bisphosphatase 1 (FBP1), and fatty acid synthase (FASN), according to the findings of Li et al. (152) in his quest to identify metabolism-related protein biomarkers of insulin resistance.

### 7.1 Biomarkers for disease progression and complications in individuals with diabetes using metabolomics

Various research has shown that when the diabetic disease condition progresses, several complications set in, including but not limited to diabetic neuropathy, diabetic kidney disease (DKD), diabetic retinopathy, and CVDs (153), Some of the metabolites discovered as biomarkers in the early progression of diabetic kidney disease in the blood serum of T2D patients, according to the study carried out by Balint et al. (153), includes arginine, dimethylarginine, hippuric acid, indoxyl sulfate, butenoylcarnitine, and sorbitol, and p-cresyl sulfate was found in the urine of the patients. Previous research has also shown that indoxyl sulfate content in serum and urine is elevated in DKD patients (154). Furthermore, the levels of p-cresyl sulfate were found to be dependent on creatinine concentrations in the blood (153, 155).

Together, these metabolites shift show that metabolic dysregulation extends beyond glucose control but progressively reflects organ-specific injury, particularly in the kidney. This makes metabolite panels a useful tool not only for early detection of diabetic complications but also for tracking disease progression over time.

## 8 Nutritional and therapeutic applications of metabolomics

Metabolomics has become an important tool in diabetes research. This is because of its ability to capture how the body responds to food at the molecular level, especially through small molecules like amino acids, lipids, and organic acids (156). Researchers are able to go beyond traditional markers like glucose and HbA1c to detect early metabolic disturbances linked to insulin resistance and β-cell dysfunction (157). Diabetes patients can also benefit from early nutritional assessment by identifying shifts in their metabolism even before the appearance of clinical symptoms. Again, these metabolic signatures also help identify dietary biomarkers and make it easier to objectively assess food intake and understand why people respond differently to the same diet (158). Consequently, metabolomics supports the transition from general dietary advice to precision nutrition. In precision nutrition, dietary recommendations are tailored to an individual’s metabolic profile, thereby improving glycemic control and reducing the risk of progression of diabetes (159).

From a therapeutic point of view, metabolomics has found significant application in biomarker identification, and this helps predict diabetes risk and monitor patients’ responses to interventions over time (78). Many human studies have consistently linked key metabolites, such as branched-chain amino acids, aromatic amino acids, acylcarnitines, diacylglycerols, and ceramides, with insulin resistance and poor glycemic control as shown on Table 1. These metabolites are being explored as potential clinical markers to track disease progression and evaluate the effectiveness of lifestyle and drug-based interventions (160). Additionally, metabolomic profiling can also help distinguish different metabolic phenotypes of T2D (161). Hence, it has become possible to match patients with more targeted dietary or pharmacological strategies and shift diabetes management toward a more individualized model where treatment is guided by metabolic patterns instead of a one-size-fits-all approach.

Therefore, metabolomics connects nutrition, metabolism, and therapy in a single framework and is becoming more important in diabetes care. As the analytical methods continue to evolve, it is expected to play a stronger role in early risk detection, dietary stratification, and monitoring therapeutic outcomes. Overall, these findings suggest that metabolomics will remain central to the development of precision nutrition and personalized diabetes therapy in the coming years.

## 9 Limitations of the study and future perspectives

### 9.1 Limitation of the study

This study evaluates advances in metabolomic technology, including super-resolution MS and NMR spectroscopy, as well as improvements in tools used for data analysis and integration (162). It also highlighted some of the biomarkers identified in the early onset and disease progression of IR, T1D and T2D (145, 162, 163). However, one of the limitations seen in this study includes variability in sample size and methods used for various biomarker identification study designs, without a universal standard method. This heterogeneity could result in non-uniformity in the use of specific metabolite results, and result in reproducibility challenges (163).

Another limitation is technological constraints that make the annotation of unknown metabolites challenging. Although the use of advanced technological equipment is continuously evolving, research shows that less than 11% of these metabolites are likely identifiable in the mass spectral archive, when using MS, for instance (164, 165). This means that about 90% of the metabolites left cannot be identified, and these could include loss of new and important metabolites that could be used as key biomarkers in the early identification and management of DM (24, 164).

Complex data analysis and interpretation are other limitations in the metabolic profiling of DM studies. The complexity of the data could be attributed to the variability or differences in analytical technologies used. Complications from the use of these instruments, such as instrument errors during analysis, can also lead to variability in data set (24, 166).

### 9.2 Future perspectives

For future studies, more work needs to be done in integrating artificial intelligence and other omics technologies in identifying biomarkers more accurately in DM prediction. More studies need to be done in expanding dietary intervention trials using metabolic endpoints, to create more personalized nutrition intervention (34). There is also a need for devising a more standardized biomarker identification method to minimize variability in biomarker identification and metabolomic data (163, 164).

## 10 Conclusion

Conclusively, this study was able to point out the key roles of metabolomics in enhancing broader knowledge, as well as early detection, risk stratification and management of DM. Findings from this study demonstrates that metabolic profiles in DM show several alterations in major metabolite classes which included the BCAA, AAA, and lipid related metabolites such as ceramides, that are positively associated with IR and DM progression. It also shows the importance of novel biomarkers such as coded microRNAs, and glycerophospholipids in early identification of DM. These metabolites are especially important for identifying prediabetes and stratifying individuals with high risk of developing diabetes, and therefore, keeping up with disease progression as well as personalized nutrition. Though the use of advanced technological methods, such as NMR, LC-MS, GC-MS, and CE-MS, has been utilized in improving metabolites detection and biomarker identification, there is a need to address the ongoing or persistent challenges, including method standardization and data variability. There is also a need to devise more efficient ways of applying metabolomics in assessing and personalizing dietary interventions for diabetic conditions. Therefore, continued research is needed to advance diabetes metabolomics profiling into a sustainable solution for both public health and global well-being.

## Data Availability

The datasets analyzed during this study are available from the authors upon reasonable request. Requests to access these datasets should be directed to John O. Onuh, jonuh@tuskegee.edu.

## Glossary

AAA: Aromatic amino acids
ATP: Adenosine triphosphate
BCAA: Branched chain amino acids
CE-MS: Capillary Electrophoresis Mass Spectrometry
DAG-Diacylglycerol
DKD: Diabetic Kidney Disease
DM: Diabetes mellitus
FADH: Flavin Adenine dinucleotide
FASN: Fatty acid synthase
FBP1: Fructose-1,6-bisphosphatase
GCK: Glucokinase
GC-MS: Gas Chromatography Mass Spectrometry
GD: Gestational diabetes
IR: Insulin resistance
LC-MS: Liquid Chromatography Mass Spectrometry
NADH: Nicotinamide Adenine dinucleotide
NMR: Nuclear Magnetic Resonance
T2D: Type 2 diabetes
TCA-Tricarboxylic Acid
TAG: Tricaylglycerol
PC: Phosphatidylcholines
PE: phosphatidylethanolamines
